# Features of Changes in the Structure and Properties of a Porous Polymer Material with Antibacterial Activity during Biodegradation in an In Vitro Model

**DOI:** 10.3390/polym16030379

**Published:** 2024-01-30

**Authors:** Vladimir V. Yudin, Tatyana I. Kulikova, Alexander G. Morozov, Marfa N. Egorikhina, Yulia P. Rubtsova, Irina N. Charykova, Daria D. Linkova, Maya I. Zaslavskaya, Ekaterina A. Farafontova, Roman S. Kovylin, Diana Ya. Aleinik, Sergey A. Chesnokov

**Affiliations:** 1 Privolzhsky Research Medical University of the Ministry of Health of the Russian Federation, 10/1, Ploshchad Minina i Pozharskogo, 603005 Nizhny Novgorod, Russia; 2 Laboratory of Photopolymerization and Polymer Materials, G. A. Razuvaev Institute of Organometallic Chemistry, Russian Academy of Sciences, 49, Tropinina, 603950 Nizhny Novgorod, Russia

**Keywords:** antibiotic release, porous polymer, ethylene glycol dimethacrylate, polylactide, porosity, cell adhesion, scanning electron microscopy, photopolymerization, scaffolds

## Abstract

Hybrid porous polymers based on poly-EGDMA and polylactide containing vancomycin, the concentration of which in the polymer varied by two orders of magnitude, were synthesized. The processes of polymer biodegradation and vancomycin release were studied in the following model media: phosphate-buffered saline (PBS), trypsin-Versene solution, and trypsin-PBS solution. The maximum antibiotic release was recorded during the first 3 h of extraction. The duration of antibiotic escape from the polymer samples in trypsin-containing media varied from 3 to 22 days, depending on the antibiotic content of the polymer. Keeping samples of the hybrid polymer in trypsin-containing model media resulted in acidification of the solutions—after 45 days, up to a pH of 1.84 in the trypsin-Versene solution and up to pH 1.65 in the trypsin-PBS solution. Here, the time dependences of the vancomycin release from the polymer into the medium and the decrease in pH of the medium correlated. These data are also consistent with the results of a study of the dynamics of sample weight loss during extraction in the examined model media. However, while the polymer porosity increased from ~53 to ~60% the pore size changed insignificantly, over only 10 μm. The polymer samples were characterized by their antibacterial activity against *Staphylococcus aureus*, and this activity persisted for up to 21 days during biodegradation of the material, regardless of the medium type used in model. Surface-dependent human cells (dermal fibroblasts) adhere well, spread out, and maintain high viability on samples of the functionalized hybrid polymer, thus demonstrating its biocompatibility in vitro.

## 1. Introduction

The last decade has been characterized by the widespread use of porous polymers both in various production industries [1,2,3,4,5,6,7,8,9,10] and in medical practice [1,11,12]. The medical field’s growing interest in porous polymers is due to the increasing need for osteoplastic materials [13,14,15]. This is related to the high number of bone defects resulting from degenerative diseases, severe injuries and the active application of surgical treatment approaches in orthopedics, oncology, and maxillofacial surgery. Antibacterial properties are a very important advantage of such materials for use in bone grafting, because the possibility of infection associated with the installation of the graft is one of the most serious problems for various areas of current high-tech surgery [16]. Currently, a lot of research is being conducted to solve such problems [17,18,19,20]. To obtain polymers with antibacterial properties, the addition of antibiotics or metal nanoparticles to the polymer or polymerizing composition is actively used. At the same time, it is very difficult to obtain a material that exhibits antibacterial properties in the required time interval. To solve this problem, we proposed an original approach using a hybrid porous polymer material containing biologically inert poly(dimethacrylate) and bioresorbable polylactide (PLA). Previous studies [2] demonstrated the possibility of creating a non-toxic polymer material functionalized with antibiotics, which maintained (according to the results of in vitro experiments) its bactericidal activity for 7 days. The authors used a porous hybrid polymer material consisting of a biologically inert porous polymer matrix and a bioresorbable polymer—polylactide containing an antibiotic—that covered the surface of the matrix pores. It was assumed that the identified effect was achieved due to two interrelated processes: a partial biodegradation of the polylactide and gradual antibiotic escape into the medium.

The term “biodegradation” refers to the process of the gradual destruction of materials that is directly related to the biological activity of the medium. The rate of biodegradation is a critical characteristic of every biomaterial that is to be used for biomedical applications, for instance, as scaffolds and/or grafts. In the human body, such degradation is ensured by various mechanisms: physical (dissolution), chemical (hydrolysis) and biological (enzymatic breakdown, or interaction with cells of the immune system). Each of these mechanisms is of the utmost importance for the biodegradation of a particular material and different methods can predominate at specific stages of the process. Active biodegradation is associated with enzymatic action of biological catalysts. In the case of tissue damage, such catalysts as serine proteases (trypsin, plasmin, etc.) and matrix metalloproteinases (collagenases, etc.) [21,22] are activated. Activation of these enzymes in the damage area results in the destruction of the damaged tissues and, thus, stimulates cleaning from the wound of the products of protein degradation, enabling the development of new tissues.

When modeling the biodegradation processes in vitro, various media that imitate natural body fluids are used. Usually, these are phosphate buffer solutions, growth media used to culture cells—for passive biodegradation, and enzyme solutions (for example, trypsin) to study active biodegradation. Assessment of the rate of biodegradation in model experiments is usually conducted either by determining the residual mass of samples at various stages of the study [11,12], or by determining the changing concentrations of the components of the degrading material released into the medium.

In order to identify an ideal material/scaffold, it is useful to compare the rates of polymer degradation and the provision of space for new tissue with the rate of new tissue formation [3,4]. Thus, each of the body tissues requires a certain rate of biomaterial biodegradation. In the case of materials intended for bone tissue replacement, the location of the defect is also important, given the particular structural features of the specific bone for which use of this material is intended. For example, one should consider that the rate of biodegradation of porous materials is, in general, greater than of non-porous ones, and that it also depends on the pore size. This is due to the fact that not only liquid media but also enzymes can penetrate into the material through its pores. As a result of such enzyme diffusion within the liquid media, destruction therefore occurs throughout the material, and not only on its outer surface [5,6].

In the abovementioned studies [2], non-toxic materials with antibacterial activity containing broad-spectrum antibiotics (vancomycin and gentamicin) were obtained, and pure cultures of various strains of staphylococci were used to demonstrate the bactericidal activity of the material samples. The positive results have spurred continued research in this sphere.

This article presents for the first time the results of a study aimed at determining the mechanism of antibiotic release from a hybrid polymer and, accordingly, determining ways to control the process. The study concentrated on the following: (i) the impact of the antibiotic content in the polylactide layer on the antibiotic concentration when it enters the model medium and on the kinetics of its release; (ii) the impact of the properties of the model medium on this process; (iii) assessment of the changes in the porous polymer monolith during this process; and (iv) determination of the impact of the properties of the medium on the bactericidal activity and cytocompatibility of the material.

The porous polymer monolith used in the study was a well-proven [2] material made from a porous matrix of polyEGDMA with a layer of polylactide applied to its surface. A broad-spectrum antibiotic, vancomycin, was used, the concentration of which incorporated into the polylactide differed by two orders of magnitude at the start of the experiment. Experiments were conducted in the following three model media: phosphate-buffered saline (PBS), trypsin-Versene solution, and trypsin-PBS solution. During the study, the dynamics of the antibiotic release from the polymer in each of the model media were monitored using the high-performance liquid chromatography (HPLC) technique, to determine the changing concentration of vancomycin in the media throughout the duration of the experiment. Simultaneously, a range of studies were conducted on changes in the properties of the medium (pH measurements), together with changes in the bactericidal activity of the polymers (disc diffusion test) and of the polymer characteristics using gravimetry, porometry and scanning electron microscopy (SEM).

## 2. Materials and Methods

*Materials*: Ethylene glycol dimethacrylate (EGDMA) (98%, Aldrich, St. Louis, MO, USA), 1-butanol (BuOH) (99.5%, Aldrich, St. Louis, MO, USA) and vancomycin (Kraspharma JSC, Krasnoyarsk, Russia) were each used without further purification.

*Model media*: Phosphate-buffered saline (PanEco LLC, Moscow, Russia); 0.25% trypsin solution (Sigma Aldrich, Darmstadt, Germany) mixed with Versene solution (PanEco LLC, Moscow, Russia), and 0.25% trypsin solution (Sigma Aldrich, USA) mixed with PBS (PanEco LLC, Moscow, Russia).

The Versene solution is a solution of the sodium salt of ethylenediaminetetraacetic acid in PBS.

*Synthesis of porous monoliths*: Porous polymers were made in line with the technique in [23]. The photopolymerizing composition was prepared from a mixture of EGDMA and 1-butanol in a weight ratio of 30:70. The finished composition was put into a mold formed of two flat silicate glasses with a 4 mm filler. Exposure started one minute after putting the composition into the mold. A Philips UHP halogen lamp (400–750 nm, 190 W) was used to initiate polymerization. The composition was exposed for 2 h to an illumination level of I = 50 kLx on the surface of mold.

*Samples of hybrid porous polymers* were obtained in line with a known procedure [2]. For that, polylactide with a molecular weight of 20 kDa was used. The synthesis of polylactide has been previously described [24].

*The pore characteristics* of the hybrid porous polymer monoliths (average pore size, D_mod_, porosity, ε) were determined by mercury porometry using a PASCAL EVO 140/440 (Thermo Scientific, Rodano, Italy). The preliminary cut samples of porous polymers of 0.1–0.2 g in weight were kept in a vacuum cabinet for at least three hours at room temperature. Then, the samples were put in a glass dilatometer and analyzed. The pore size was identified within the range from 3.6 nm to 120 μm, which corresponded to a pressure range of 10 Pa–400 MPa applied to the sample.

*Scanning electron microscopy:* SEM examination of slices of the porous polymer monoliths was performed using a Regulus SU8100 microscope (Hitachi, Tokyo, Japan). The sizes of the pores and polymer globules were assessed on the basis of the SEM images obtained.

*The dynamics of changes in pH* of the model media and the properties of the hybrid polymer samples in the model media were determined as follows. Weighed and labeled samples of the hybrid polymers of 8 × 8 × 4 mm were put in the wells of 24-well plates (Costar, Washington, DC, USA) filled with 2.0 mL of the relevant medium per sample and placed in a CO_2_ incubator at t = 37 °C, 5% CO_2_ and absolute humidity. Incubation conditions were constantly monitored. At test periods (3 h, then 1, 3, 7, 10, 14, 21, 28, 37 and 44 days), three polymer samples from each series were taken, and the medium was sampled for analysis. After each experiment test period, the pH of the solutions and the weight of each polymer sample were determined, in addition to assessment of the samples’ pore characteristics and their surface structure.

*The pH values of the media* were determined using a “Checker” portable pH meter (HANNA instruments, Romania).

*Gravimetric studies* were conducted using an HTR-220CE analytical balance (ViBRA, Ibaraki, Japan), with an accuracy of 1 mg. PLA weight loss (%) = ((m_1_ − m_2_)/(m_1_ − m_0_)) × 100, where m_0_—mass of the original matrix; m_1_—mass of the matrix with a PLA layer; m_2_—mass of the matrix with a PLA layer after exposure to a model environment.

*The content of polylactide or polylactide with vancomycin* in the hybrid polymer samples was determined gravimetrically by the difference in the samples’ masses before and after soaking them in solutions of polylactide, or polylactide with vancomycin, in 1,4-dioxane. The weight loss of the polylactide (polylactide with vancomycin) in the sample after exposure to the model medium was determined by the difference in the sample mass before and after exposure of the sample to the model medium. The relative weight loss of the polylactide (polylactide with vancomycin) after exposure to the model medium was calculated by dividing the weight loss of the sample by the initial weight of polylactide (polylactide with vancomycin) in the sample. The relative weight loss was determined for three samples at each period of exposure to the model medium.

*The dynamics of vancomycin release* from the polymer samples into the model medium and the dependence of this process on the vancomycin concentration were assessed by preparing samples of the hybrid polymer (of size of 8 × 8 × 4 mm) with different antibiotic concentrations: 10%, 1%, and 0.1%. These samples were filled with model media—2 mL per sample—and placed in the wells of a 24-well plate (Costar, Washington, DC, USA). Before the experiment, the pH of the solutions was measured: phosphate-buffered saline (PBS)-7.49; 0.25% trypsin-PBS solution-7.40; 0.25% trypsin-Versene solution-7.38.

The plate with samples was placed in a CO_2_ incubator and maintained under standard conditions (37 °C, 5% CO_2_, absolute humidity). After 3 h of incubation, the extract above the samples was completely removed to determine the vancomycin content. Then the samples were filled with fresh model media—2 mL per sample. Twenty-four hours after the beginning of the experiment, the medium above the samples was removed and the wells were filled with fresh media—2 mL per sample. After 3 h of further incubation, the extracts were sampled to determine the antibiotic content. At each of the following test periods of 3, 7, 10, 14 and 21 days after the beginning of the study, the wells were emptied and refilled with appropriate medium, which was then sampled after three hours of extraction, and the antibiotic content therein was determined using HPLC. Each series of examinations was conducted in triplicate. Thus, the authors determined the capacity of samples with varied antibiotic content to release it into the medium over three-hour periods after predetermined periods of biodegradation in the different model media.

*Analysis of model media for their vancomycin content* was conducted by HPLC on a Knauer liquid chromatograph (Germany) with a UV detector at wavelengths of 280 nm and 210 nm, on a 6 × 100 mm steel column filled with Separon Si C18 sorbent with a particle size of 5 μm. Elution was conducted in isocratic mode with a methanol–aqueous solvent (+0.05 mM trifluoroacetic acid) with a ratio of 30:70 by volume. Quantitative calculations were based on the absolute calibration method using calibration solutions of vancomycin hydrochloride in the eluent, prepared in bulk on an HTR-220CE analytical balance (ViBRA, Ibaraki, Japan) to an accuracy of 1 mg. Quantitative processing of the chromatograms was performed using MultiChrome 1.5 software (Ampersend).

*The samples’ bactericidal activity* was determined by the disk diffusion method [25,26]. The study was conducted with a pure culture of a clinical isolate of *Staphylococcus aureus* (strain 191), which had shown high sensitivity to vancomycin [27]. The staphylococci were cultivated (24 h, 37 °C) on beef-extract agar (Federal Budget Institution of Science “State Research Center for Applied Microbiology and Biotechnology” (FBUN SRCAMB), Obolensk). The bacterial suspension seeded onto the plates had previously been prepared in phosphate-buffered saline (PBS) (pH 7.2–7.4; “PanEco”, Moscow, Russia) with a 0.7 concentration according to McFarland. The bacterial suspension (0.05 mL) was distributed over the agar surface to achieve confluent seeding of the staphylococci.

Discs with vancomycin (5 μg, HiMedia Laboratories Pvt. Ltd., India) were used as control samples—sample No. 1). During the preparation of the polymers functionalized with 5 μg of vancomycin, the samples were put in separate sterile containers with 2 mL of liquid: phosphate-buffered saline—sample No. 2, 0.25% trypsin-PBS solution—sample No. 3 or 0.25% trypsin-Versene solution (pH 7.2–7.4; “PanEco”, Moscow)—sample No. 4. The exposure of the polymers functionalized with vancomycin (samples Nos. 2, 3, 4) was conducted in closed containers in the corresponding model media for 3 h, or for 1, 3, 7, 14 and 21 days. After these times, the samples were removed with sterile tweezers and placed on the surface of the staphylococcus cultures growing on beef-extract agar in a Petri dish. A standard paper disk with vancomycin (control) was simultaneously placed onto the same culture. The cultures were further incubated for 24 h at 37 °C, and then the diameter (in mm) of the bacterial growth inhibition zone around the test samples was measured. Each series was conducted in triplicate.

*Adhesion and viability of cells on the surfaces of the samples.* Data from previous studies demonstrated good adhesion and proliferation of mesenchymal stem cells and surface-dependent human cells on various porous polymer monoliths [7,28] and hybrid polymer samples without any antibiotic [29]. However, material functionalization with an antibiotic can lead to changes in its biological characteristics. The authors demonstrated that the inclusion of vancomycin in a hybrid polymer in an amount of 10% or less did not impact the material, and it remained non-cytotoxic. Complete assessment of the cytocompatibility of the material functionalized with vancomycin was conducted on the basis of the determination of the extent of adhesion and viability of human cells on the material samples. The assessment of each material’s biological properties was conducted using test cultures of human dermal fibroblasts of 3–4 passages. The cultures were obtained and characterized in the biotechnology laboratory of the University clinic of the FSBEI HE PRMU of the Russian Ministry of Health. The cultures used were sterile and uncontaminated with mycoplasmas and viruses. The cell viability of the cultures before the experiment was 97–98%. Fibroblasts with a density of 20,000/cm^2^ were seeded onto the surfaces of 3 samples of the hybrid porous polymer containing vancomycin in 2 mL of complete growth medium. The complete growth medium was Dulbecco’s modification of Eagle’s medium with the addition of antibiotics (penicillin/streptomycin), 2% glutamine and 10% fetal calf serum (PanEco LLC, Moscow, Russia). After 24 h, the samples were removed, stained with fluorescent dyes, and then visualization of the cells on the surface of the samples was conducted.

To visualize the cells that had adhered to the surface of the opaque material samples, cell nuclei were intravitally stained with the fluorochrome Hoechst 33342 (USA, excitation wavelength of 377 nm and emission wavelength of 447 nm), which is specific to double-stranded DNA molecules. The cells’ cytoplasm was visualized using Calcein AM (BD Pharmingen™, with an excitation wavelength of 495 nm and emission wavelength of 515 nm). The principle of Calcein AM action is based on the esterase activity typical only of viable cells. The fluorochrome staining was performed according to the manufacturers’ protocols.

Fluorescence microscopy was implemented using a Cytation 5 imager (BioTek, Winooski, VE, USA) with Gen 5 Image+ software, with the microphotographs then stored in a video archive.

## 3. Result and Discussion

### 3.1. Wancomycin Release

**Figure 1:**
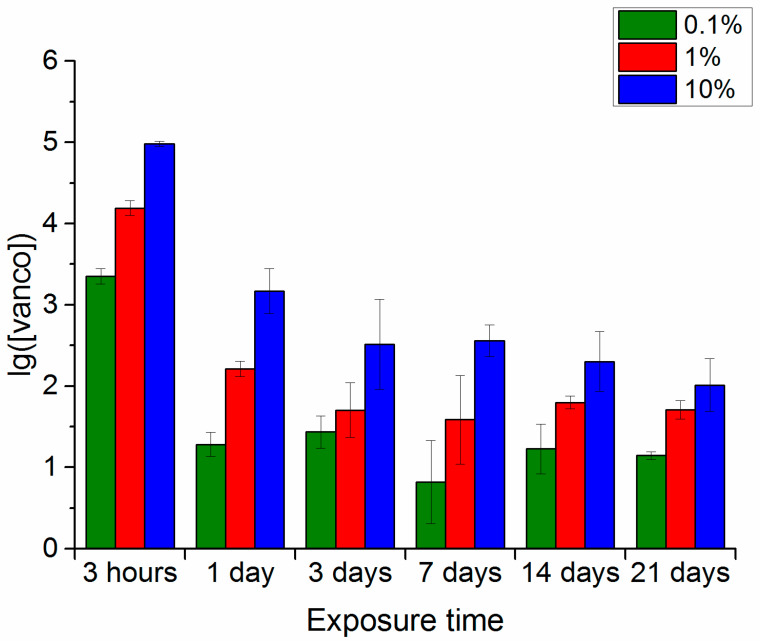
Change in vancomycin content in the polymer sample extracts (accumulated over 3 h extraction periods) with different drug contents, depending on the overall exposure time in phosphate-buffered saline.

**Figure 2:**
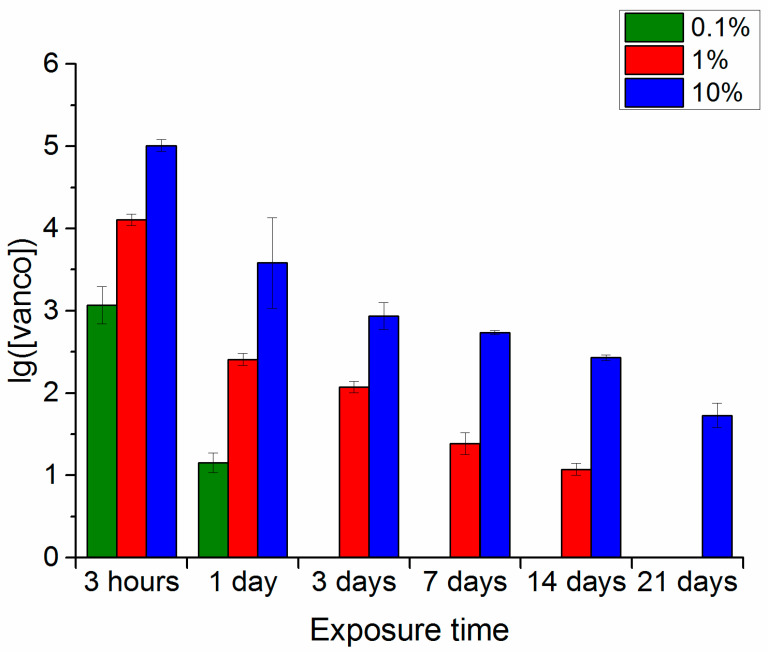
Vancomycin content in extracts of polymer samples with different concentrations of the antibiotic when kept in a trypsin-Versene solution and sampled at the specified intervals.

**Figure 3:**
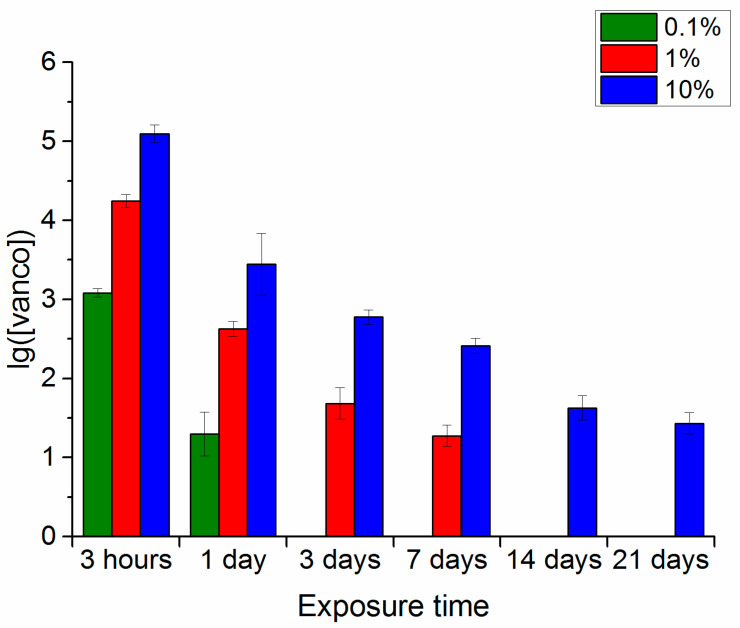
Vancomycin content in extracts of polymer samples with different drug contents when kept in the trypsin-PBS solution and sampled at the specified intervals.

The results of assessing the dynamics of vancomycin release from the hybrid porous polymer samples with different antibiotic concentrations in phosphate-buffered saline, 0.25% trypsin-Versene solution and 0.25% trypsin-PBS solution are shown in Figure 1, Figure 2 and Figure 3, respectively. Analysis of the data on vancomycin release into phosphate-buffered saline from the polymer samples containing 0.1, 1 and 10% vancomycin (Figure 1) indicates the following. Regardless of the drug concentration in the samples, it was released from the samples at all test points up to and including 21 days (Figure 1). Increased exposure time to the different media resulted in the antibiotic release from all samples consistently decreasing. In general, the antibiotic content in the extracts at each test point in the study decreased by 1.2–3 times compared with the initial antibiotic content in the samples, even though the starting concentrations had been two orders of magnitude different.

Substitution of the phosphate-buffered saline with the trypsin-Versene solution led to a significant change in the resulting the antibiotic concentration in the extracts over the duration of the experiment. The main differences were seen for an exposure time of 3 days and, as with the phosphate-buffered saline, the maximum content of vancomycin was seen in the extracts of all samples 3 h after the beginning of the experiment (Figure 2). After 24 h, the concentration of vancomycin released into the extracts had decreased, and this decrease was more pronounced than for the phosphate buffer. After 3, 7 and 14 days of extraction, the antibiotic could be identified only in extracts of samples originally containing 10% and 1% vancomycin. After 21 days of biodegradation, the antibiotic remained detectable only in extracts of samples with an initial maximum drug concentration of 10%.

In the experiment with the solution of trypsin in PBS, as in previous cases, the maximum content of the antibiotic in the medium for all samples was seen 3 h after the beginning of extraction (Figure 3). After 24 h, the antibiotic could still be determined in the extracts of all samples under study. For the polymer samples with 10 and 1% vancomycin, the antibiotic content in the extracts decreased in a quite similar fashion to that in the phosphate-buffered saline. For the polymer sample with 0.1% vancomycin, at 24 h, the antibiotic concentration in the extract was lower than in the phosphate-buffered saline. After three and seven days of extraction, the antibiotic could be determined only in extracts from the samples containing 10 and 1% vancomycin. After 14 and 21 days of the experiment, the antibiotic was detectable only in extracts of samples with the maximum initial drug concentration of 10%.

Thus, the duration and intensity of the broad-spectrum antibiotic (vancomycin) extraction from the polymer samples depended on the drug concentration in the samples and the nature of the medium in which the extraction was conducted. The longest period for maintaining detectable drug extraction (up to 21 days) from the polymer samples was recorded for its maximum concentration of 10% in all model media. With a drug concentration of 1%, the possibility of extraction continued for a long time in the phosphate-buffered saline, persisted up to 14 days in the trypsin-Versene solution, and up to 7 days in the trypsin-PBS solution. With the minimum concentration of 0.1% vancomycin in the polymer samples, the drug was identified in extracts during the entire experiment only in the phosphate-buffered saline. In all other media, it could not be detected after 3 days. However, one should note that the maximum drug release was recorded in the first 3 h after exposure of the samples to the model media and, here, the vancomycin content in the extracts essentially did not differ, regardless of the medium type used for extraction.

In line with the previously proposed process model [2], vancomycin was released into the medium as a result of decomposition of the polylactide layer on the surface of the polymer sample. This assumption was verified by studying changes in some of the properties of the polymers and the characteristics of the model solutions during the experiment.

### 3.2. Change in pH of Model Media

**Figure 4:**
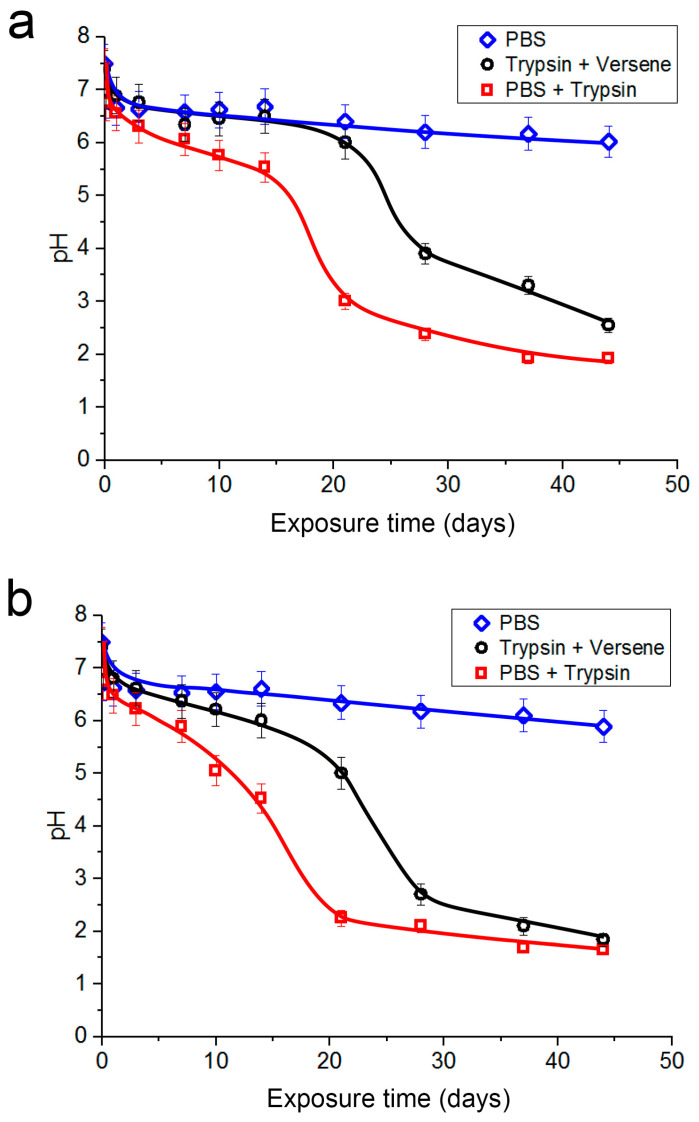
Change in the pH of the model media of the samples depending on the time of exposure: (**a**) samples without vancomycin; (**b**) samples with 10% vancomycin.

The polylactide decomposition product is lactic acid. Thus, when the hybrid polymer is exposed to the model media, the polylactide decomposes, which should lead to a decrease in the pH of the medium. Figure 4a shows the pH change curves for the model media over 44 days in contact with the polymer samples with a polylactide coating containing vancomycin 10%. The initial pH values of the solutions were 7.4–7.5. In all cases, by 3 h after the beginning of the experiment, a sharp decrease in the pH of the solutions was recorded: by 0.7 in phosphate-buffered saline, by 0.4 in the trypsin-Versene solution, and by 0.65 in the trypsin-PBS solution. Subsequently, acidification of the medium in the phosphate-buffered saline was very slow, and by day 44, the pH was 6.02 (blue curve). The solutions with trypsin showed a step-like dependence of the pH of the medium on the exposure time (red curve), a pattern that was present but less pronounced for the trypsin-Versene solution (black curve). Here, the first stage of a slow decrease in pH from 6.98 to 6.0 continued for 21 days. Then, the pH decreased to 3.9 during the next 7 days and, after that, it slowly (over the subsequent 16 days) decreased to 2.55. In the trypsin-PBS solution (red curve), there was a gradual decrease in pH from 6.76 to 5.53 over the first 14 days; the pH then dropped sharply to 3.0 over the next 7 days and, after that, the pH decreased to 1.93 during the next 23 days.

Similar dynamics in the acidity changes of the medium were seen when studying a batch of samples of the hybrid polymer containing vancomycin and placed in the corresponding model media (Figure 4b). Here, too, an insignificant decrease in pH was detected in the phosphate-buffered saline (blue curve); the release of vancomycin developed at the highest speed in the trypsin-PBS solution (red curve) and a little less fast in the trypsin-Versene solution (black curve). At the same time, there were a number of differences. Firstly, in the trypsin-containing media, the process was faster during the first stage, where the pH level began to decrease immediately after the beginning of the experiment. Secondly, the final pH values in these media were also lower: 1.84 in the trypsin-Versene solution and 1.65 in the trypsin-PBS solution compared to 2.55 and 1.93, respectively, for the polymers without vancomycin.

A comparison of the dependences of the extracts in model media on the vancomycin content of the polymer sample (10% vancomycin) and the changes in the pH of the model media over the duration of incubation of the hybrid polymer samples with vancomycin (also 10%) indicated the following. Phosphate-buffered saline—compared to other media, both processes were the slowest. Trypsin-Versene solution—the time dependence curve of vancomycin release (Figure 2) showed an initial sharp release of the drug, but this rate of vancomycin release had then decreased by the third day, only to accelerate again during the period of 14–22 days of exposure. With trypsin-PBS solution, the curve of dependence of pH on the experiment time (Figure 4b) also showed that the decrease in pH became faster after 14 days of exposure. Here, the rate of vancomycin release from the samples was higher than that in the trypsin-Versene solution, with it increasing after 7 days of the experiment, meaning that the pH of the solution also decreased sharply after 7 days of exposure.

Thus, keeping samples of the hybrid polymer in the model trypsin solutions led to acidification of the solutions, evidencing biodegradation of the polylactide in the samples. This, in turn, resulted in leaching of the vancomycin into the studied medium. Trypsin, like other serine proteases, is a catalyst for hydrolytic destruction of polylactide, although the activity of the enzyme is known to be significantly reduced in the presence of the sodium salt of ethylenediaminetetraacetic acid, which is a component of the Versene solution [30]. This accounts for why the polylactide degradation in the presence of the trypsin-PBS medium was faster than in the trypsin-Versene solution. In contrast, as there is no enzyme in the phosphate-buffered saline medium, the polymer decomposition is much slower, as the accumulation of lactic acid formed during hydrolysis during the initial stage (potentially reaching pH 4.5), leading to inhibition of the process [31].

The rate of change in the pH level of the model media surrounding the polymer samples also correlates well with the data on autocatalysis during the hydrolytic decomposition of PLA in [31]. The curves demonstrating the changes in pH of samples exposed to trypsin-PBS solution or the trypsin-Versene solution system (Figure 4) have three sections with different slopes. For instance, during the initial stage, the rate of pH change in trypsin-PBS was 0.1 pH units /day, whereas the trypsin-Versene solution system slowed down the process by half (0.05 pH units/day). When pH values of 4.5–5 had been passed, an increase in the rate of acidification to 0.30–0.35 pH units /day was seen, with the rate here unaffected by the presence of the trypsin inhibitor, as the PLA hydrolysis, accompanied by the free acid accumulation, was influenced by the protons of the acid. At the last stage, due to the predominance of the random mechanism of the polymer chain destruction, the decrease in pH slowed down (0.03–0.05 pH units/day). With vancomycin, hydrolysis during the initial stage proceeded with a faster accumulation of protons (−0.16 pH units/day or −0.09 pH units/day with trypsin-Versene solution system) due to vancomycin’s high acidity (pH 2.5–4.5) [32]. The second stage, characterized by the maximum change in pH (−0.33 pH units/day), had the same parameters as seen during hydrolysis of the PLA containing no antibiotic.

**Figure 5:**
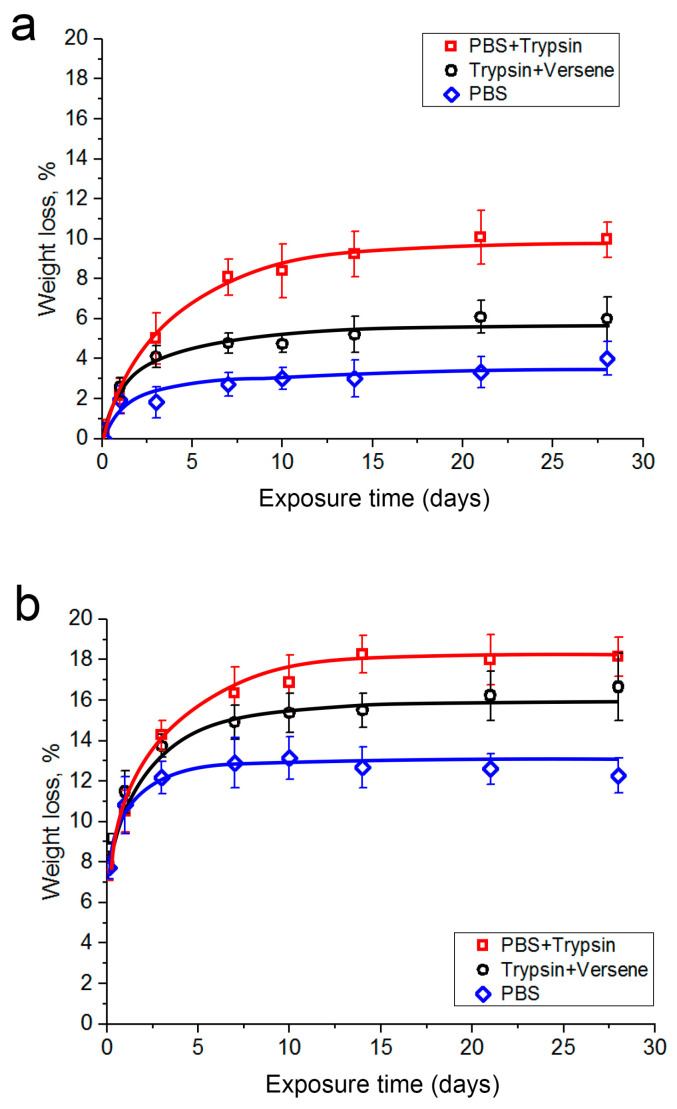
Dependence of the relative weight loss of the hybrid polymer samples (**a**) without vancomycin and (**b**) with vancomycin (10%) on the duration of the samples’ exposure to the model solutions: (**a**) weight losses from samples relative to the weight of polylactide in the samples; (**b**) weight losses from samples relative to the total weight of polylactide and vancomycin in the samples.

Figure 5 provides graphs demonstrating the loss in weight of the hybrid polymer samples when the samples were kept in the model media. Figure 5a shows data for samples without vancomycin. The curves were built with the assumption that the weight loss of each sample was related to the polylactide weight in the sample before the experiment. Figure 5b shows data for samples with vancomycin (10%). The curves here were built with the assumption that the weight loss of each sample was related to the total weight of polylactide and vancomycin in the sample before the experiment (it was established in a separate experiment that the weight of samples without polylactide did not decrease). One can see that the relative weight loss of the samples increases in both cases when considering, first, the weight loss with the phosphate-buffered saline then with the trypsin-Versene solution and, finally, the trypsin-PBS solution. In the first case (Figure 5a), the curves reached a plateau after 21 days of exposure; in the second case, the curves reached a plateau after 10 days of exposure (Figure 5b). The weight loss of samples with vancomycin was greater than that of samples without vancomycin, which may be attributable to the dissolution of the vancomycin in the medium as well as to accelerated polylactide biodegradation. This is confirmed by the faster and stronger acidification of the trypsin-containing solutions with the samples containing vancomycin (Figure 4).

Thus, the results of studying the dynamics of the weight loss of samples with and without vancomycin in the examined model media are consistent with the data on the vancomycin release from the samples (Figure 1, Figure 2 and Figure 3) and the changes in the pH levels of the solutions (Figure 4).

### 3.3. MIP and SEM Data

A critical feature of the studied hybrid polymer samples is their porosity. The pore characteristics of the samples after their exposure to the model media for 3 h and 21 and 28 days were studied to answer the question of what happens to the samples’ porous structure when they are kept in the model media. The results are provided in Table 1.

The presented data clearly demonstrate that when the PLA solution is applied to the surface of the porous matrix, the average pore size changes to larger values, whereas the value of porosity actually decreases. For instance, the pore size of the control samples increased from 9.3 μm to 11.0 μm for the hybrid polymer and to 9.8 μm for the hybrid polymer with antibiotic, while the sample porosity decreased from 69.5% to 57.9% for the hybrid polymer and to 54.6% for the hybrid polymer with antibiotic. When the samples were kept in the model media, the average sample pore size changed and was within the range of 9.8–11.7 µm for the hybrid polymer and between 9.4 and 11.5 µm for the hybrid polymer with antibiotic. Here, the porosity of the samples increased. The increase in the sample porosity can be seen in the various model media but with different extents. For example, over 28 days, the porosity increased from 57.9% to 59.1% for the hybrid material and to 59.4% in the phosphate-buffered saline and in the trypsin-Versene solution, respectively, and to 63.5% in the trypsin-PBS medium. It can be concluded that the increase in the sample porosity is related to the destruction of the polylactide layer, which is greatest in the trypsin-PBS solution. For the hybrid materials with the antibiotic, the differential impact of the nature of the model medium leveled out over 28 days: the porosity changing from 54.6% to the similar values: 60.3%, 61.3% and 59.9% for phosphate-buffered saline, the trypsin-Versene solution and the trypsin-PBS solution, respectively. It seems that the increase in the sample porosity here is equally effective due to the simultaneous destruction of the polylactide layer and dissolution of the antibiotic particles. However, by 56 days, the porosity of the samples in the trypsin-PBS solution had increased to 67.4%, while for the samples in phosphate-buffered saline and the trypsin-Versene solution, it had not changed. This is another confirmation of the greater activity of the trypsin-PBS solution.

Below is a comparison of these results with the SEM data. Three groups of porous polymers were studied. The first group included samples before exposure to the physiological medium—a porous polymer matrix before (No. 1) and after the application of the PLA layer (No. 2), as well as after the introduction of vancomycin (No. 3). The second group consisted of samples of the porous polymer materials with PLA, kept for 28 days in phosphate-buffered saline (No. 6), in the trypsin-Versene solution (No. 9) and in the trypsin-PBS solution (No. 12). The third group included samples of the porous polymer materials with PLA and vancomycin, kept for 28 days in phosphate-buffered saline (No. 15), in the trypsin-Versene solution (No. 19) and in the trypsin-PBS solution (No. 23).

**Figure 6:**
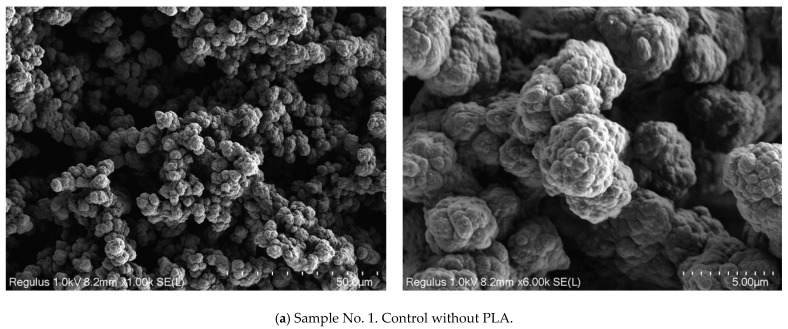

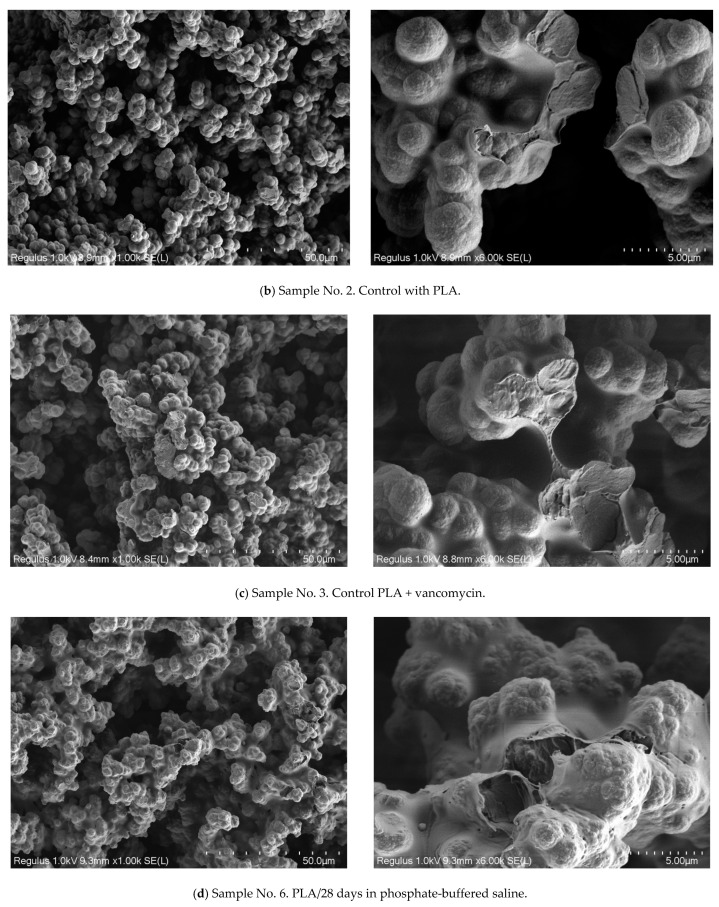

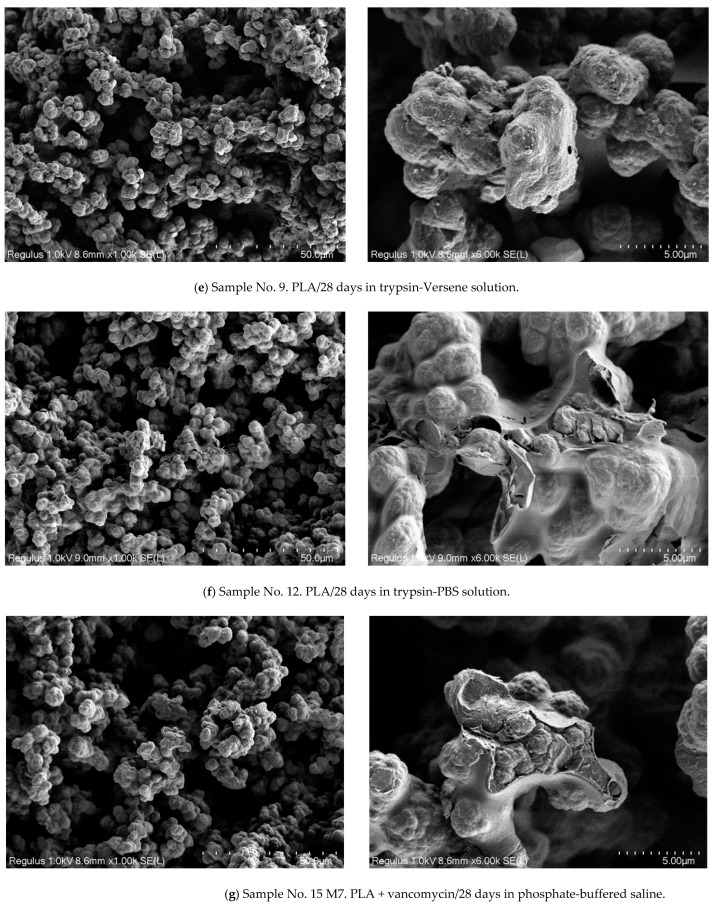

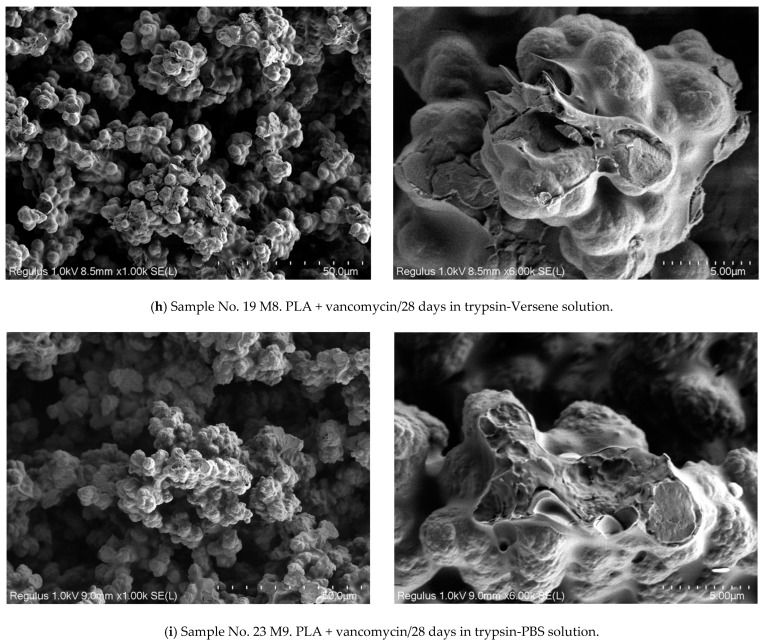
SEM images of fractures: samples before exposure to a physiological medium—porous polymer matrix (**a**) before (sample No. 1) and (**b**) after treatment with the PLA solution (sample No. 2), as well as (**c**) after treatment with the PLA solution with vancomycin (sample No. 3); samples of porous polymer materials treated with the PLA solution and kept in a phosphate-buffered saline for 28 days (**d**) (sample No. 6); in the trypsin-Versene solution (**e**) (sample No. 9) and a mixture of phosphate buffer and trypsin (**f**) (sample No. 12); samples of porous polymeric materials treated with the PLA solution with vancomycin and kept in phosphate-buffered saline for 28 days (**g**) (sample No. 15), in the trypsin-Versene solution (**h**) (sample No. 19) and the trypsin-PBS solution (**i**) (sample No. 23).

Figure 6 shows microphotographs of fractures of porous polymer materials M1–M9. Below is a detailed consideration of the first group of samples Nos. 1–3, which were not exposed to the model media. The structure of the porous polymer of sample No. 1 before treatment with the PLA solution is a system of interconnected agglomerates of spherical particles with sizes 0.3–0.5 µm, looking like coral (Figure 6a). The size of the agglomerates is up to 3–5 µm. Between the particle clusters, there is a continuous free space, which is a system of interconnected pores. The size of the agglomerates can be flexibly adjusted and is determined by the conditions of synthesis of the porous polymer—that is, by the polymerizing composition, temperature, and intensity of the initiating radiation. The smallest cavities in the contact areas of the spherical particle agglomerates have a size of ~1 μm. In general, a system of interconnected agglomerates of spherical particles form the walls of pores of various shapes, and with a diameter of ~10–12 μm, which is consistent with the results of the mercury porometry (Table 1).

Comparison of microphotographs of sample No. 1 and sample No. 2—after the application of PLA to its surface (Figure 6b)—identifies that a PLA film has appeared on the polymer agglomerates, which makes the surface of the globules smoother. Analysis of microphotographs of sample No. 2 also shows that the boundaries between the spherical particles of the agglomerates disappear, although the agglomerates of 3–5 μm are still visible. The microphotographs also make it clear that the thickness of the PLA film is 0.1–0.2 microns, and the macropores are not closed by the polylactide; thus, the system of interconnected open pores is still available. Hence, coating the pores with polylactide, essentially, does not change the structure of the pores or their size. Similar conclusions can be made based on analysis of the SEM images of sample No. 3 (Figure 6c) with vancomycin. It is seen that the structure of sample No. 3 is similar to that of sample No. 2—there being no vancomycin particles on the polymer material, even though it contains the antibiotic.

Analysis of microphotographs of samples Nos. 6, 9 and 12, that is, the results of exposing the original porous polymers with PLA to the various model media for 28 days, indicates the following: Figure 6d–f makes it clear that the open pore structure and the PLA layer on the surfaces of the pores have been preserved in all three samples. The images obtained at a magnification of 6000× show that the PLA layer has become thinner and the microstructure of the spherical particle agglomerates of the polymer matrix appears more pronounced compared to the original sample No. 2.

The SEM study of the third group of samples Nos. 15, 19 and 23 (Figure 6g–i) with vancomycin, which were kept for 28 days in the various model media, showed that these samples, as in series Nos. 6, 9 and 12, have an open pore structure and a PLA layer on the surface of the pores. Additionally, compared to the original sample No. 3, there is a noticeable decrease in the PLA layer, while the microstructure of the spherical agglomerates is more pronounced.

### 3.4. Bactericidal Activity of Vancomycin Immobilized on the Surface of the Hybrid Material Pores

Vancomycin is a drug whose properties allow it to be introduced into grafts and medical materials and is effective in the local prevention and treatment of periprosthetic infection [33]. Thus, the purpose of introducing the antibiotic into the porous hybrid polymer is to ensure its antibacterial activity. Hence, an important characteristic of such a polymer is the dynamics of any changes in the bactericidal activity of vancomycin immobilized on the pore surfaces during long-term exposure of the polymer, as tested using the model media. The antibacterial activity of vancomycin in the polymer was assessed using samples of 10% vancomycin, as these samples demonstrated the longest period (21 days) of detectable antibiotic release into all the model media.

The bactericidal activity of samples with vancomycin was determined via the disk diffusion method [27,34] using a vancomycin-sensitive strain of *Staphylococcus aureus* [25,26]. Polymer samples with 10% vancomycin were kept in the model media for 3 h, 1, 3, 7, 14 and 21 days and were then put in Petri dishes of staphylococcus cultures; after 24 h of exposure at 37 °C, the diameter of the bacterial growth inhibition area around the studied samples was measured. Diagnostic discs with vancomycin (HiMedia Laboratories Pvt. Ltd., India) were used in the same dishes as a reference.

**Figure 7:**
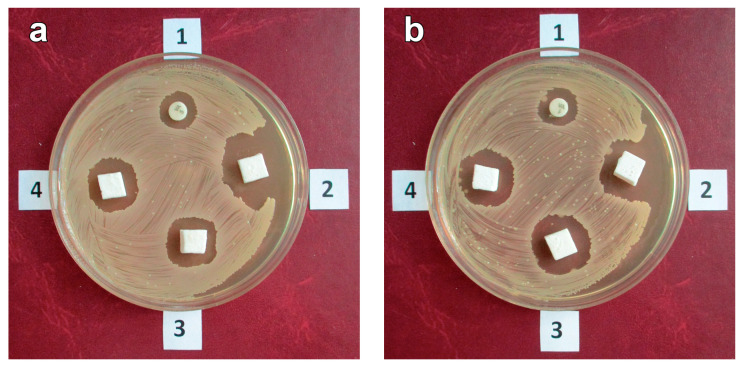

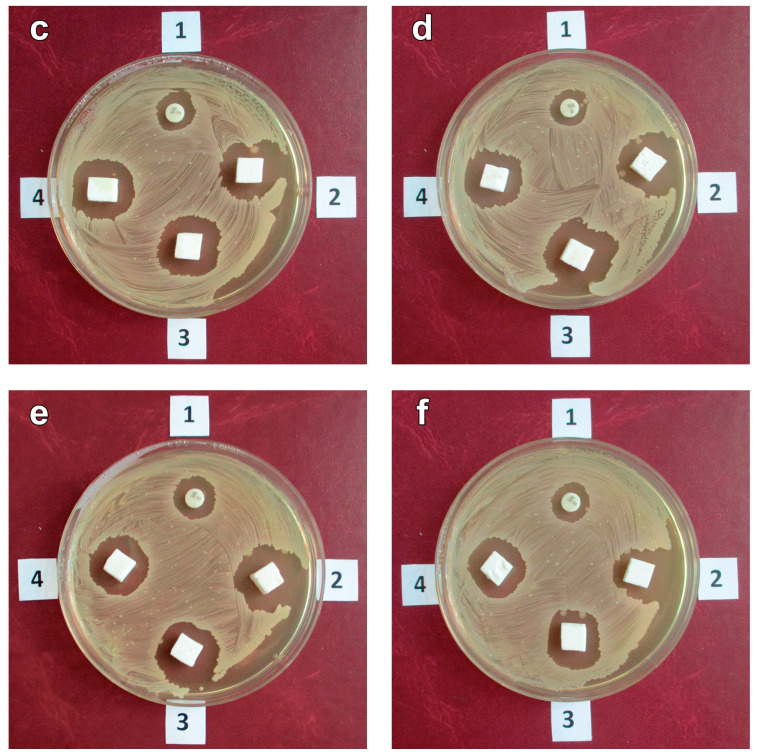
The sizes of the growth inhibition areas on staphylococcus test cultures around samples of the control disk with vancomycin (No. 1) and samples of the hybrid polymer with a vancomycin concentration of 10% after immersion in phosphate-buffered saline (No. 2), in the trypsin-PBS solution (No. 3) and in the trypsin-Versene solution (No. 4), depending on the time in the medium: 3 h (**a**), 1 day (**b**), 3 days (**c**), 7 days (**d**), 14 days (**e**) and 21 days (**f**).

Figure 7 provides photographs of the Petri dishes with the polymer samples, originally with 10% vancomycin, after the test exposure periods.

The data in Figure 7 demonstrate that 10% vancomycin, immobilized on the surface of the hybrid polymer pores, retains its expressed bactericidal activity during the entire study period (21 days), after incubation with all of the model media. Therefore, one can consider that samples with vancomycin incubated in different media do not fundamentally differ in their antibacterial activity, regardless of the polymer exposure time in the medium. The results suggest that the action of proteolytic enzymes in the area of material implantation should not fundamentally impact the antibiotic’s antibacterial activity when a graft made of the studied porous polymer hybrid material is implanted into the body.

### 3.5. Adhesion and Cell Viability on the Surface of the Porous Polymer with Antibacterial Activity (10% Vancomycin)

**Figure 8:**
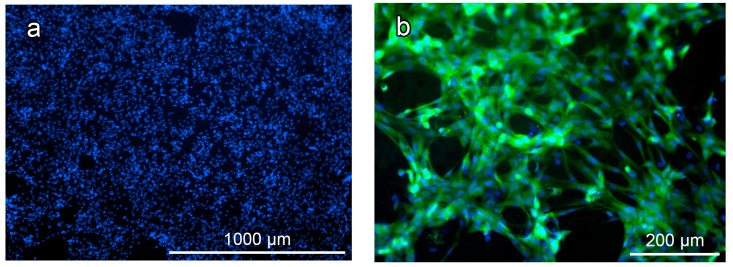
Fibroblasts on the surface of the hybrid polymer samples: (**a**) fibroblast nuclei on the surface of the hybrid polymer samples, stained blue (Hoechst fluorochrome); (**b**) fibroblasts spread on the surface of the hybrid polymer samples: the cell cytoplasm is stained green (Calcein AM fluorochrome), and cell nuclei are blue (Hoechst fluorochrome).

Previously, the authors demonstrated good adhesion and proliferation of cells on the surface of a hybrid polymer that did not contain antibiotic [29]. The data in Figure 8a show that surface-dependent cells (human dermal fibroblasts) also adhere well to the surface of the hybrid polymer containing vancomycin. Indeed, our use of calcein flurochrome (Calcein AM), which stains the cytoplasm only of viable cells, allowed us to visualize the viable (labeled with calcein) spindle-shaped cells of typical fibroblast-like shape, which were spread over the entire surface of the samples (Figure 8b).

Thus, the study recorded the capacity of the test culture cells to adhere well and spread out in a formation with a typical morphology, as well as to maintain their viability on the functionalized hybrid polymer samples. The results prove the cytocompatibility of the studied hybrid material and give grounds to consider that the developed hybrid material is promising for research and biomedical use.

## 4. Conclusions

The method suggested herein to immobilize the vancomycin antibiotic on the surfaces of the pores of the hybrid polymer material allows loading of the target drug in a wide range of concentrations differing by up to two orders of magnitude. Samples containing 0.1 and 1% vancomycin in media with trypsin showed time-limited antibacterial activity—a maximum of 14 days. However, the porous hybrid material with 10% vancomycin retained local antibacterial activity for at least 21 days in the in vitro model in all the model media used. Here, the duration of continued, detectable antibiotic diffusion into the medium depended on both the antibiotic concentration and the medium type. It has been shown that biodegradation is caused by biodegradation of the polylactide layer, this being confirmed by changes in the acidity of the medium, the dynamics of the weight reduction of the samples and the visual decrease in the polylactide layer (according to the SEM results) from the surfaces of the pores in the samples. Despite this, it was demonstrated that during the 28 days of observation, the porosity of the polymer samples changed only insignificantly in all the model media. Consequently, changes in the material porosity and its bactericidal activity are not related to the type of model medium or the duration of polymer exposure to the medium. The results suggest that the action of proteolytic enzymes in the area of polymer implantation should not fundamentally affect the porous characteristics of the material or the activity of an antibiotic (such as vancomycin) contained within it when a graft made from the material under study is implanted into the body. This assumption will be verified in further preclinical studies of the material using an in vivo model. A positive property of the polymer used is its cytocompatibility, as demonstrated by the interaction of human cells with it in vitro. The material’s porous structure, its antibacterial properties and cytocompatibility give grounds to consider this type of polymer material as a basis for the further development of a new generation of grafts for the treatment of bone defects.

## Figures and Tables

**Table 1:** Pore characteristics of samples of the porous polymer matrix alone, the porous hybrid polymer without an antibiotic and with vancomycin (10%) before and after exposure to the model media.

№	Conditions		Time, Days	D_mod_, μm	ε, %
1	Control without PLA		0	9.3	69.55
2	Control with PLA		0	11.0	57.97
3	Control with PLA + vancomycin		0	9.8	54.56
4	PLA	PBS	0.125	11.0	62.03
5	PLA	PBS	21	9.8	58.12
6	PLA	PBS	28	11.7	59.10
7	PLA	Trypsin + Versene	0.125	10.4	57.17
8	PLA	Trypsin + Versene	21	11.2	61.88
9	PLA	Trypsin + Versene	28	10.7	59.35
10	PLA	Trypsin + PBS	0.125	11.0	61.53
11	PLA	Trypsin + PBS	21	11.2	64.28
12	PLA	Trypsin + PBS	28	11.3	63.49
13	PLA + vancomycin	PBS	0.125	9.5	62.25
14	PLA + vancomycin	PBS	21	9.5	62.70
15	PLA + vancomycin	PBS	28	10.8	60.27
16	PLA + vancomycin	PBS	56	9.5	57.23
17	PLA + vancomycin	Trypsin + Versene	0.125	9.8	61.58
18	PLA + vancomycin	Trypsin + Versene	21	9.5	61.67
19	PLA + vancomycin	Trypsin + Versene	28	10.3	61.32
20	PLA + vancomycin	Trypsin + Versene	56	10.5	60.66
21	PLA + vancomycin	Trypsin + PBS	0.125	11.5	59.63
22	PLA + vancomycin	Trypsin + PBS	21	11.2	56.94
23	PLA + vancomycin	Trypsin + PBS	28	9.4	59.97
24	PLA + vancomycin	Trypsin + PBS	56	10.7	67.35

## Data Availability

The raw/processed data required to reproduce these findings cannot be shared at this time as the data also form part of an ongoing study.
